# Evaluation of progress toward universal health coverage in Myanmar: A national and subnational analysis

**DOI:** 10.1371/journal.pmed.1003811

**Published:** 2021-10-15

**Authors:** Zlatko Nikoloski, Alistair McGuire, Elias Mossialos

**Affiliations:** Department of Health Policy, London School of Economics and Political Science, London, United Kingdom; Harvard University, UNITED STATES

## Abstract

**Background:**

Universal health coverage (UHC) encompasses 2 main components: access to essential healthcare services and protection from financial hardship when using healthcare. This study examines Myanmar’s efforts to achieve UHC on a national and subnational level. It is a primer of studying the concept of UHC on a subnational level, and it also establishes a baseline for assessing future progress toward reaching UHC in Myanmar.

**Methods and findings:**

The study uses the Demographic and Health Survey (2015) and the Myanmar Living Conditions Survey (MLCS; 2017) and adapts a previously developed UHC index to provide insights into the main barriers preventing the country’s progress toward UHC. We find a negative correlation between the UHC index and the state/region poverty levels. The equity of access analysis reveals significant pro-rich inequity in access to all essential healthcare services. Socioeconomic status and limited availability of healthcare infrastructure are the main driving forces behind the unequal access to interventions that are crucial to achieving UHC by 2030. Finally, financial risk protection analysis shows that the poor are less likely to use healthcare services, and, once they do, they are at a greater risk of suffering financial catastrophe. Limitations of this study revolve around its correlational, rather than causal, nature.

**Conclusions:**

We suggest a 2-pronged approach to help Myanmar achieve UHC: Government and state authorities should reduce the financial burden of seeking healthcare, and, coupled with this, significant investment in and expansion of health infrastructure and the health workforce should be made, particularly in the poorer and more remote states.

## Abstract 2

Zlatko Nikoloski and colleagues provide analytical insight into Myanmar’s efforts to achieve universal health coverage on a national and sub-national level.

## Author summary

**Why was this study done?:**

- The UN Sustainable Development Goal (SDG) 3 encompasses achieving universal health coverage (UHC), including financial risk protection, access to quality healthcare services, and access to safe, effective, quality, and affordable essential medicines and vaccines for all. More specifically, SDG Target 3.8 stipulates achieving UHC, including financial risk protection, access to quality essential healthcare services, and access to safe, effective, quality, and affordable essential medicines and vaccines for all.
- While to date, cross-country progress toward UHC has been gauged by constructing indices, to the best of our knowledge, no such index has been constructed on a subnational level, particularly in the context of a large and heterogeneous country such as Myanmar.

**What did the researchers do and find?:**

- We adapt a previously developed UHC index, and we apply it to the context of Myanmar while using the most recent nationally and subnationally representative data.
- Our analysis yields robust links between socioeconomic development and UHC at subnational level in Myanmar.
- More importantly, our analysis also suggests significant pro-rich inequities in access to essential healthcare services, which are driven by both wealth and availability of healthcare.
- Finally, we find robust evidence that the poor, in addition to having a more limited access to healthcare, are more likely to spend higher share of their domestic budgets on healthcare, thus having a greater risk of suffering a financial catastrophe.

**What do these findings mean?:**

- There is a significant heterogeneity in UHC progress in Myanmar broadly reflecting socioeconomic disparities across states and regions.
- Progress toward UHC could be achieved by investing in healthcare as well as by adopting protective measures that would reduce the financial burden of seeking healthcare.
- Our results could provide a benchmark for assessing Myanmar’s efforts toward UHC, particularly given the uncertainties with the recent political turmoil.

## Introduction

Universal health coverage (UHC) encompasses 2 main components: access to essential healthcare services and protection from financial hardship when using healthcare [1]. In line with the Sustainable Development Goals (SDGs) Agenda 2030 of “leaving no one behind,” the aim of UHC is to promote equitable access to essential healthcare services [2]. To date, progress toward UHC has been gauged by constructing indices that include both UHC components mentioned above [3–5].

Some of these indices have been used in global cross-country comparative analyses [3,4,6]. Furthermore and in the context of East and Southeast Asia, the concept of UHC has been analyzed extensively, and studies point to large cross-country differences, particularly in terms of financial risk protection [7]. However, while some Southeast Asian countries (e.g., Thailand and Vietnam) have been extensively studied in terms of progress toward UHC, there is very little relevant evidence on Myanmar [7]. Myanmar has featured in the global UHC index, but, to date, no such index has been constructed on a subnational level [4]. This is particularly relevant for Myanmar—a large, heterogeneous, and federative state. Constructing a UHC index on a subnational level in the context of Myanmar could, therefore, be a primer that could be applied in similar settings across the globe and, in particular, in “failing states.” After 10 years of economic transformation, on February 1, 2021, the country’s military staged a coup, effectively seizing the country’s commanding heights. Applying the UHC index could, therefore, provide a baseline for further analysis of the socioeconomic impact of a regime change.

Myanmar is a lower middle-income country, which registered a per capita gross domestic product (GDP) of $5,142 USD in purchasing power parity (PPP) terms in 2019 [8]. With a total population of 54 million, Myanmar is the fourth most populous country in Southeast Asia (after Indonesia, the Philippines, and Vietnam) [8]. The country can be divided into 5 physiographic regions—the northern mountains, the western ranges, the eastern plateau, the central basin and lowlands, and the coastal plains [9]. Beginning in 2011, Myanmar undertook a journey of triple transitions: toward peace in the border areas; a move to democratic governance; and through the establishment of a nascent market-oriented economy [10]. The ensuing economic reforms and trade deregulation resulted in rapid growth at an annual average of 7%, among the fastest in the East Asia-Pacific region and globally [8]. The acceleration in GDP growth was followed by a significant reduction in poverty rates, from 48.2% in 2005 to 24.8% in 2017, when using the national poverty line as a benchmark [11].

Since it emerged from its years of isolation, Myanmar has scaled up its efforts to achieve UHC. More recently, its government adopted the National Health Plan (NHP) (2017 to 2021) to strengthen the healthcare system and set out a path toward achieving UHC. The aim of the NHP is to extend access to a basic “Essential Package of Health Services” (EPHS) to the entire population while gradually increasing financial protection [12]. Consistent with WHO’s objectives, the EPHS prioritizes primary healthcare and the delivery of essential services at the township/village level and below, starting within the community [13].

Myanmar’s healthcare system has undergone significant changes due to recent reforms to the country’s political and administrative structure, and, currently, there are health departments at regional/state, district, and township levels [14,15]. Existing evidence has identified several challenges facing the healthcare system. First, there is chronic underfunding of the healthcare sector. Public spending on health has been persistently low over the last 20 years, teetering around 1% of GDP [8]. Second, the lack of funding has resulted in notable shortages of human resources for health. In particular, general practitioners (GPs) and nurses/midwives number 0.68 and 0.99 per 1,000 people, respectively—falling well below the regional averages of East Asia (1.5 and 2.6 per 1,000 people, respectively) [8]. Third, the distribution of healthcare infrastructure, such as buildings and equipment, is unequal. According to the latest available data, there are 0.9 hospital beds per 1,000 people in the country (on average), which is far lower than the regional average of 3.5 [8]. Moreover, there is a historical mismatch between health maps of the administrative units mentioned above and catchment areas of health facilities, leading to challenges in estimating catchment population [12]. Fourth, in terms of service provision, there is an emphasis on secondary and tertiary care, with facilities at lower levels receiving much less attention. This has been further exacerbated in the conflict-prone and post-conflict areas, where there is limited public sector service delivery, and most of the services are provided by nongovernmental organizations or the private sector. More broadly, the existing evidence points to substantial public/private segmentation of healthcare provision, with poor oversight of the private sector, where services are variable and often of questionable quality [13]. Fifth, health insurance is available to government employees, but not to the majority of the population. Coupled with the overall low spending on healthcare, this implies that a majority of healthcare spending is out of pocket. The latest data indicate that 76.2% of health financing is out of pocket, which is much higher than the regional average of 36.1% [8].

Some of these challenges and bottlenecks at the national level have implications for the availability and distribution of healthcare facilities at the subnational (state/union) level. A WHO review of Myanmar’s healthcare systems reveals that poor states such as Rakhine or Ayeyawady have fewer beds and healthcare facilities per 1,000 population compared to the national average [16]. Furthermore, there is lower density of hospital beds in states with remote areas such as Chin and Kayah. This is mostly driven by the fact that hospitals are allotted according to geographical terrain to cover people in the physically hard-to-reach areas with sparse population in those states [16]. On the other hand, the availability of healthcare facilities and density of hospital beds are highest in both Myanmar’s commercial capital (Yangon) and in its administrative capital (Nay Pyi Taw) [16]. These disparities in the availability of healthcare infrastructure closely match the broader socioeconomic disparities across the states/regions in Myanmar. The most recent poverty estimates indicate that half of the population in Chin and Rakhine live below the national poverty line [17]. This is mirrored in other indicators of socioeconomic development, such as education attainment rates or access to clean water and improved sanitation [11]. These socioeconomic inequities undoubtedly have consequential impacts on the overall access to and utilization of healthcare services across states and regions.

These shortcomings have implications for both aspects of UHC, service provision and financial risk protection, at the national and subnational level. Against this background, our main objective is to provide a comprehensive and up-to-date assessment of Myanmar’s progress toward UHC on both the national and subnational levels. Moreover, our study represents a primer for constructing a UHC index on a subnational level that could be applied in other large, heterogeneous, and federative countries.

## Methods

This study is a secondary data analysis of existing datasets, and, as such, it did not include prospective protocol. The analyses and the dataset are described further below. Given the richness and comprehensive nature of the datasets used, no data-driven changes to the analyses took place.

### UHC index

In order to assess Myanmar’s progress toward UHC, we adapted Wagstaff and Neelsen’s index, which captures both dimensions of UHC: service coverage (everyone, irrespective of ability to pay and getting the services they need) and financial protection (nobody suffering financial hardship as a result of receiving needed care) [4,5]. The values of the index run from 0 to 100, with higher scores being better in terms of progress toward achieving UHC [5]. There are 3 reasons why constructing this index is an attractive option. First, it has already been applied to a wider set of 111 countries globally [4]. Second, the approach has been used by the World Bank in constructing a database on progress toward UHC [6]. Finally, as previously pointed out, the proposed index combines the merits of the “dashboard” approach and the overall index approach [5,18].

We used this adapted index for both national and subnational analyses of the states and regions of Myanmar. The set of indicators and their definitions are listed in Table A in S1 Tables and include 4 of Wagstaff and Neelsen’s set of prevention and treatment indicators (i.e., 4 antenatal care visits, full immunization, skilled birth assistance and healthcare sought for common childhood illnesses, and inpatient healthcare use among the adult population) as well as their indicator capturing financial risk protection (i.e., catastrophic healthcare expenditure [CHE]) [4]. We have excluded 2 of the prevention indicators used in the original index—cervical cancer screening and breast cancer screening as there are no data available for these indicators on the subnational level in Myanmar. As an interesting aside, only one of these (cervical cancer screening) is featured in an alternative index, because of clearer guidelines for cervical cancer and because cervical cancer screening is the only cancer screening indicator included in the core indicator set of the noncommunicable diseases global monitoring framework [3]. Nevertheless, this alternative index contains a much wider set of indicators that go beyond health coverage and financial risk protection (e.g., access to sanitation, international health regulations, and antiretroviral therapy [ART] treatment for HIV patients). Hence, we opted for following the approach by Wagstaff and Neelsen [4].

All of the indicators that we used are expressed as coverage (i.e., the share of population using the service), except for 2: those for financial protection and inpatient admissions. The financial protection indicator is equivalent to 100 minus the percentage of the population incurring CHE—i.e., expenditure in excess of 10% (or 25%, in a sensitivity analysis) of their overall household level consumption. The inpatient admissions indicator we normalized at the population level using the WHO benchmark of 0.1 inpatient admissions per capita, equivalent to 9.03% of the population with an inpatient admission in the past 12 months [19].

The final index, then, is a geometric mean of the indicators capturing the 2 dimensions of UHC: financial protection and service coverage, where both dimensions are weighted equally. The service coverage dimension is a geometric mean of 2 domains: prevention and treatment, which are weighted unequally (25% on prevention and 75% on treatment), based on the share of spending on both preventative and curative services [4]. The prevention domain itself is a geometric mean of 2 indicators: at least 4 antenatal care visits and full immunization, both of them weighted equally. Finally, the treatment domain is a geometric mean of 4 indicators: skilled birth assistance, care seeking for acute respiratory infection, care seeking for diarrhea, and inpatient admissions, weighted unequally according to the share of expenditure on inpatient versus outpatient healthcare services (Table A in S1 Tables) [4]. The index was calculated on a national and subnational level and correlated with the poverty headcount rate that has been previously reported elsewhere [11].

### Statistical analysis

In addition to adapting and creating the UHC index on a national and subnational level, we conducted statistical analyses on service coverage and financial protection indicators in order to shed further light on the key problems preventing Myanmar from achieving UHC.

We conducted an equity analysis of the UHC index service coverage indicators. More specifically, for this set of indicators, concentration index (CI) and decomposition of the CI exercise was conducted in order to (i) estimate the national and subnational level of unequal access to selected coverage indicators; and (ii) to further assess the main contributors to this unequal access [20]. The CI used here is associated with certain shortcomings, such as the “bounds issue” for bivariate variables, i.e., equal concentration indices of 2 countries that have different mean rates of utilization of a given service reflect different levels of inequity in access, because “the mean of the distribution places bounds on the possible values of the concentration index” [21]. Hence, caution should be exercised when using the index for cross-country and intertemporal comparison, and, for this purpose, various adjustments to the CI have been proposed [21,22]. For our study, however, we only focus on one country in one point in time. In the decomposition analysis, a battery of independent variables was used to capture enabling factors (e.g., educational attainment and wealth index) as well as community level factors (urbanicity and region of residence) (further details are provided in S1 Text). In particular, the decomposition analysis helped shed further light on some of the main barriers to making access to selected interventions more equal.

Given the shortcomings of equity analysis in relation to the financial protection indicators, when analyzing the main drivers of CHE, we used standard logit modeling [23]. Details of the CHE calculations are provided in S1 Text. We also took into account expenditure for transport while seeking care (to the best of our knowledge, this is the first study to do so). This is important, given the low density of available healthcare infrastructure mentioned above. In the analysis, we focused on 2 main variables: socioeconomic status (as assessed by quintiles of household consumption), capturing the household’s capacity to pay; and the community module of the Myanmar Living Conditions Survey (MLCS), a proxy for availability of healthcare facilities (a binary variable capturing whether there is a healthcare facility in the village/ward or whether the nearest facility is deemed “too far”). We also controlled for the standard set of household correlates (further details are reported in S1 Text). However, it is important to note that some poor households may delay seeking care when they cannot afford to pay for healthcare out of pocket, and, thus, their health expenditure would be identified as zero, and they would not be captured as those incurring catastrophic health expenditure [24]. To control for this potential problem of sample selection associated with the fact that households can only incur CHE if they actually seek and purchase healthcare, we adopted Sartori’s 2-step approach to modeling the link between CHE and its correlates [25]. According to this approach, first, the decision to seek healthcare is modeled, followed by the modeling of the probability of incurring CHE (further details in S1 Text). In addition and in order to provide further nuance to the CHE results, we conduct a correlation analysis between regional level CHE and regional level demand variables (e.g., poverty rates, share of household members below the age of 5, and share of household members over the age of 65) as well as regional supply variables (i.e., share of communities per state/region with available primary or secondary healthcare infrastructure). This study was reported in accordance with the REporting of studies Conducted using Observational Routinely collected health Data (RECORD) statement (see S1 Checklist).

### Datasets

We relied on 2 datasets for this analysis. All of the coverage indicators (except for inpatient visits) came from the Demographic and Healthcare Survey (DHS) conducted in 2015 to 2016. Data from the survey consisted of responses from women of reproductive age living in 13,260 households, for urban and rural areas, and for each of the 7 states and 8 regions of Myanmar [26]. Data for CHE and inpatient admissions came from the MLCS conducted in 2017. MLCS 2017 is a comprehensive household survey conducted by Myanmar’s Central Statistical Organization of the Ministry of Planning, Finance, and Industry. The survey is representative at the national level, the state/regional level, and for the Union Territory of Nay Pyi Taw, including both urban and rural areas [11]. A total of 13,730 households were interviewed, yielding a wide range of information on how people work, how much income they earn, and how they use this income to meet the food, housing, health, education, and other needs of their families [11]. MLCS 2017 is particularly well positioned for our CHE analysis as one of the survey’s core objectives is an assessment of poverty and living conditions.

We have used these data sources for 5 reasons. First, the sampling frame of both surveys is based on the latest census in Myanmar (conducted in 2014), thus rendering them nationally representative. Second, both of these surveys have high responses rates, and, in addition to being nationally representative, are also representative of the different states and regions in Myanmar. Third, they are the most up-to-date sources of UHC-related data in the country. Fourth, they were both conducted around the same time (2015 and 2017, respectively). Finally, they include data collected around the time Myanmar embarked upon its NHP, thus making this a baseline against which Myanmar’s progress toward achieving UHC can be assessed.

## Results

**Fig 1:**
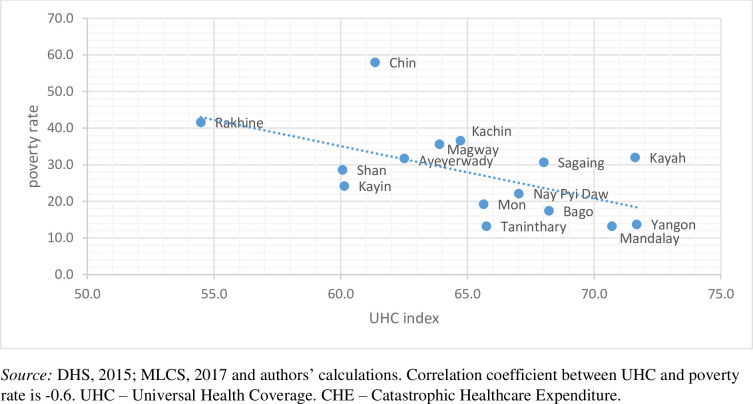
Correlation between UHC index and poverty rate in Myanmar using CHE threshold of 10%. DHS, Demographic and Healthcare Survey; MLCS, Myanmar Living Conditions Survey.

**Fig 2:**
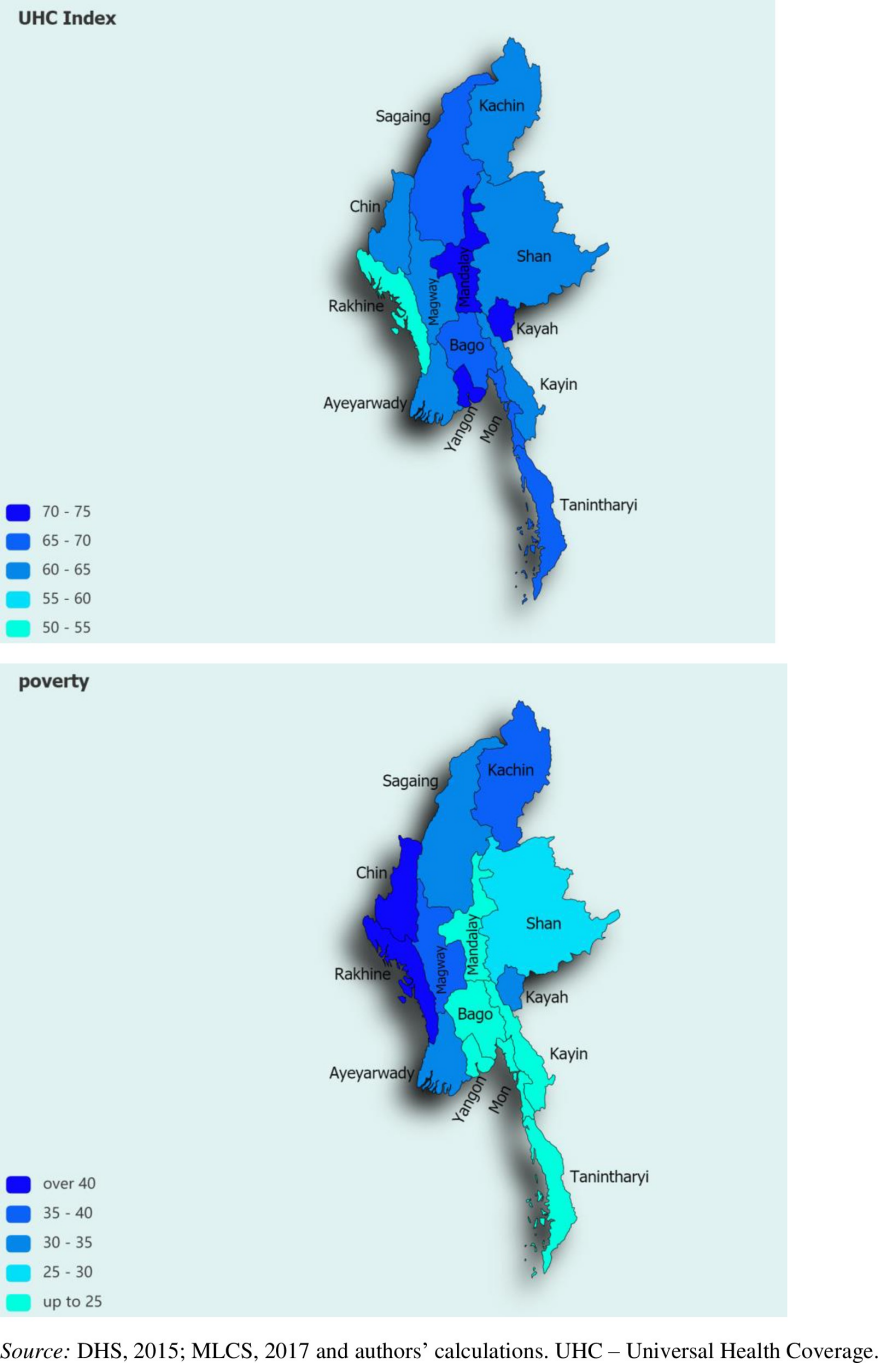
Myanmar: UHC index (top) and poverty headcount rate (bottom) on a subnational basis. DHS, Demographic and Healthcare Survey; MLCS, Myanmar Living Conditions Survey.

Table 1 presents the results of the derived UHC index at the national and subnational levels. At the national level, the index takes a value of 65.4. The table also reveals significant heterogeneity across states/regions, with higher index values found in more urban areas, such as Yangon (71.7), as opposed to some of the more rural parts of the country, like Rakhine (54.5). Table 1 also contains the disaggregated analysis of the UHC index on its main subcomponents: service coverage (both treatment and prevention) and financial protection. The disaggregated analysis confirms the heterogeneity across states/regions mentioned above, with higher service coverage (both treatment and prevention) in states like Yangon (66.3) and Mandalay (61.6) and lower service coverage in Chin (49.4). In order to emulate previous findings, which found a robust link between the UHC index and the level of socioeconomic development, we conduct a similar analysis while using poverty headcount as a proxy for subnational economic development [4]. As Fig 1 shows, there is a negative correlation between the UHC index and the state’s poverty headcount rate (correlation coefficient is −0.6). This negative correlation is further confirmed when an alternative measure of financial risk protection is used, with the correlation coefficient amounting to −0.7 when CHE at 25% threshold is used (Fig A in S1 Fig). Table 1 also shows the level of coverage for each of the indicators that make up the index. There is significant heterogeneity across states/regions of the individual index components, with coverage being higher in more urban and affluent states (e.g., Yangon and Mandalay) than in rural and less affluent states (further demographic snapshots on state/region level are provided in Table B in S1 Tables). This heterogeneity across states/regions, as well as the link between UHC and poverty level, is also evident in Fig 2.

**Table 1:** Myanmar: UHC index and coverage of selected interventions (in %), national and subnational analysis.

	Prevention		Treatment									
	Antenatal care coverage	Full immunization	Medical assistance at delivery	Diarrhea treatment	ARI treatment	Inpatient admissions index	CHE at 10%	Prevention	Treatment	Service coverage	Financial protection	UHC
Union	59.0	35.8	66.4	57.0	55.8	53.93	20.4	45.9	56.7	53.8	79.6	65.4
Urban	84.7	40.7	89.7	54.9	62.8	56.04	19.1	58.7	61.5	60.8	81.0	70.2
Rural	51.1	34.3	59.7	57.5	53.4	53.05	21.0	41.9	54.9	51.3	79.0	63.7
Kachin	61.0	43.4	70.3	56.0	51.1	38.10	13.2	51.5	47.2	48.3	86.8	64.7
Kayah	69.0	49.5	59.2	59.1	61.9	47.62	6.2	58.4	53.5	54.7	93.8	71.6
Kayin	53.8	30.1	55.3	49.1	58.2	44.85	22.7	40.2	49.2	46.8	77.3	60.1
Chin	40.2	37.5	51.5	42.1	33.6	68.44	23.7	38.8	53.5	49.4	76.3	61.4
Sagaing	54.6	45.1	73.6	55.1	62.4	60.02	20.7	49.6	61.6	58.4	79.3	68.0
Taninthary	60.3	29.4	72.8	66.2	62.2	57.14	23.0	42.1	61.8	56.2	77.0	65.8
Bago	58.6	41.8	72.8	58.7	59.1	72.31	25.6	49.5	67.6	62.6	74.4	68.2
Magway	57.3	34.9	75.2	68.8	45.6	43.41	18.2	44.7	51.8	49.9	81.8	63.9
Mandalay	67.2	39.4	85.4	63.4	69.5	59.36	18.9	51.4	65.5	61.6	81.1	70.7
Mon	64.2	42.4	74.4	67.3	75.9	47.29	24.2	52.2	58.5	56.8	75.8	65.6
Rakhine	40.6	28.1	31.0	49.2	53.6	48.50	30.2	33.7	45.9	42.5	69.9	54.5
Yangon	85.0	47.7	84.4	81.8	65.8	58.91	22.6	63.7	67.3	66.3	77.4	71.7
Shan	46.9	28.2	53.0	34.3	52.4	40.86	12.8	36.4	43.2	41.4	87.2	60.1
Ayeyerwady	57.5	23.0	55.7	64.8	56.1	47.51	18.8	36.4	52.8	48.1	81.2	62.5
Nay Pyi Daw	56.4	31.7	71.9	71.3	36.2	66.11	19.7	42.3	61.4	55.9	80.3	67.0

Source: DHS 2015, MLCS 2017, and authors’ calculations.

ARI, acute respiratory infection; CHE, catastrophic healthcare expenditure; DHS, Demographic and Healthcare Survey; MLCS, Myanmar Living Conditions Survey; UHC, universal health coverage.

In order to provide further context to the national and regional level values for the index, we reworked the original index and excluded information on cervical and breast cancer screening, as these 2 indicators are not available on state/regional levels in Myanmar (Table C in S1 Tables includes a snapshot of the results from both the old and the newly adapted version of the UHC index). The value of 65.4 places Myanmar on the 96th position, out of 111 countries from the original list. Moreover, the result is comparable to other countries in the region at similar levels of economic development (e.g., Bangladesh = 65.10 and Cambodia = 61.53). This exercise can also help us to further comment on the subnational analysis. If Yangon was an independent state, with the value of the UHC index of 71.7, it would be closer to Egypt, Pakistan, or Indonesia. On the other hand, Rakhine with a UHC index value of 54.5 is closer to places like Chad or Ethiopia (both low-income countries).

**Fig 3:**
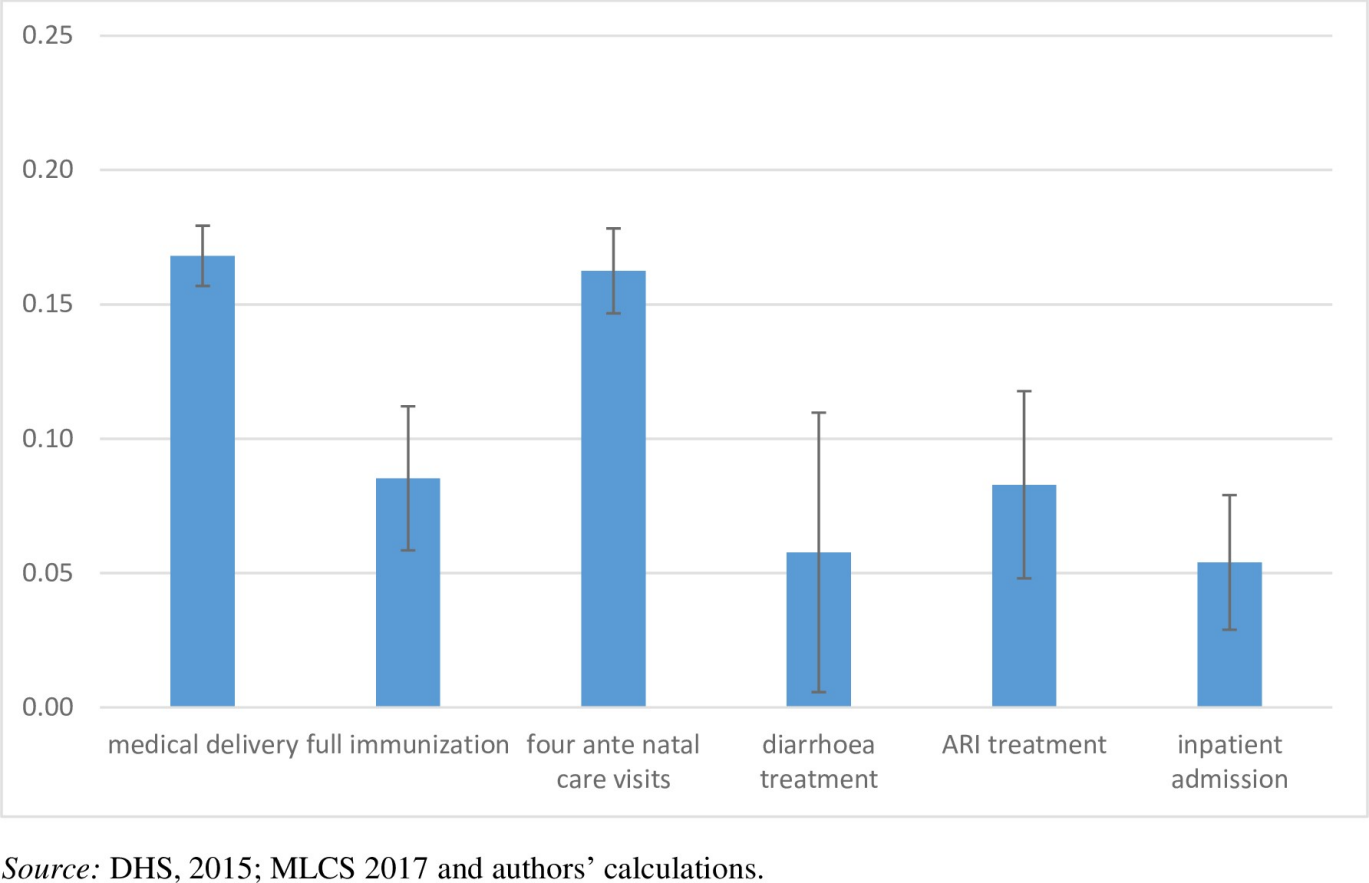
Myanmar: CI (value and 95% confidence interval) for selected prevention and treatment interventions. CI, concentration index; DHS, Demographic and Healthcare Survey; MLCS, Myanmar Living Conditions Survey.

Coverage indicators, however, often mask unequal access to selected healthcare interventions. To further investigate the equity of access to selected interventions, a CI analysis was conducted. The results of the CI analysis (Fig 3) indicate that there is a significant pro-rich inequity in access to selected prevention and treatment indicators. More specifically, the magnitude of the CI suggests particularly pronounced unequal access to antenatal care (CI = 0.16, 95% confidence interval = 0.15 to 0.18) and skilled assistance during delivery (CI = 0.17, 95% confidence interval = 0.16 to 0.18). The CI for the rest of the indicators is also positive, although smaller in magnitude, indicating a less pronounced pro-rich unequal access to the remaining interventions.

The results of the subnational level analysis are presented in Tables D and E in S1 Tables, focusing on 6 coverage indicators: at least 4 antenatal care visits, skilled assistance during delivery, full immunization, care seeking for respiratory infection, care seeking for diarrhea, and inpatient admissions. What is evident from the tables is that there is a pronounced pro-rich inequity of access for the selected interventions across all states and regions of Myanmar. Further, the results indicate a particularly pronounced pro-rich inequity in states with a higher poverty headcount. For example, in Rakhine, the CI for 4 antenatal care visits is 0.24 (95% confidence interval = 017 to 0.30), while the CI for skilled assistance during delivery is even higher, standing at 0.36 (95% confidence interval = 0.29 to 0.43). There is a similarly pronounced pro-rich inequity in poorer states for the other indicators reported in Tables D and E in S1 Tables (e.g., full immunization and care seeking for common childhood illnesses).

This exercise was coupled with a CI decomposition analysis in order to further examine the main contributors to the pro-rich inequity in access for the selected prevention and treatment indicators. The results (Table F in S1 Tables) indicate that socioeconomic status and women’s level of education are the main contributors to pro-rich inequity in access. More importantly, and potentially capturing the availability of healthcare services, urbanicity positively contributes to the pro-rich inequity of access to selected interventions. As a robustness check, we have also experimented with including the density of health workers (doctors, nurses, and midwives per 1,000) in the decomposition analysis (data on density of healthcare workers on a state/region level have been previously reported elsewhere) [27]. However, density of healthcare workers is only available at state/regional level (and therefore highly correlated with the variable capturing the 15 states/regions), which, in turn, helps to explain the findings of the decomposition analysis (Table G in S1 Tables provides a snapshot of this robustness check).

Table 2 reports the results of the logit modeling on the determinants of CHE, while focusing on socioeconomic status (captured by quintiles of household consumption) and availability of healthcare facilities (Table H in S1 Tables presents the full set of results). Overall, the findings suggest a robust link between socioeconomic status and CHE, with those in the lowest consumption quintile more likely to experience CHE than those in the other consumption quintiles. When considering the 25% threshold for CHE, for example, households in the lowest consumption quintile are 1.45 (95% confidence interval = 1.01 to 1.7) times more likely to incur CHE relative to those households in the top consumption quintile. We also found some slight evidence (only when considering the 10% CHE threshold) of a link between availability of healthcare services and probability of incurring CHE.

**Table 2:** Myanmar, determinants of CHE, logit model, ORs, and 95% confidence interval.

	10%	15%	20%	25%
**Socioeconomic status** (relative to the highest consumption quintile)				
Quintile 1	1.14 [0.91 to 1.41]	1.299** [1.00 to 1.68]	1.449** [1.05 to 1.99]	1.451** [1.01 to 1.7]
Quintile 2	0.97 [0.78 to 1.18]	0.999 [0.78 to 1.27]	0.984 [0.73 to 1.32]	1.040 [0.73 to 1.46]
Quintile 3	0.99 [0.82 to 1.19]	1.027 [0.82 to 1.28]	1.019 [0.78 to 1.32]	1.053 [0.78 to 1.41]
Quintile 4	1.053 [0.87 to 1.26]	1.169 [0.94 to 1.44]	1.133 [0.87 to 1.46]	1.059 [0.79 to 1.41]
**Availability of healthcare facilities**				
Public hospital	0.457** [0.23 to 0.90]	0.508 [0.22 to 1.16]	0.504 [0.18 to 1.37]	0.550 [0.17 to 1.74]
Public PHC	1.066 [0.88 to 1.27]	0.957 [0.77 to 1.18]	0.975 [0.75 to 1.25]	1.085 [0.81 to 1.43]
Private hospital	0.974 [0.79 to 1.18]	0.982 [0.78 to 1.23]	1.105 [0.84 to 1.44]	1.022 [0.75 to 1.37]
Private PHC	0.843* [0.69 to 1.03]	1.022 [0.81 to 1.28]	1.054 [0.80 to 1.38]	0.911 [0.67 to 1.23]
*N*	13,730	13,730	13,730	13,730
Pseudo R-sq	0.038	0.034	0.036	0.034

*** Significant at 1%.

** Significant at 5%.

* Significant at 10%.

The models also control for the following variables: age of the household head, gender of the household head, education attainment of the household head, employment status of the household head, household size, number of household members over the age of 65, number of household members below the age of 5, urbanicity (urban/rural residence) as well as regional dummies for the 15 states/regions in Myanmar. Availability of healthcare facility is derived from the community questionnaire, and it is a binary variable taking a value of 1 if there is no specified healthcare facility in the village/ward or the community does not use such facilities as they are too far. All models estimated with robust standard errors. Full set of results is reported in Table H in S1 Tables. Quintile 1 is the lowest, while quintile 4 is the highest proxy for socioeconomic status.

Source: MLCS 2017 and authors’ calculations.

CHE, catastrophic healthcare expenditure; MLCS, Myanmar Living Conditions Survey; OR, odds ratio; PHC, primary healthcare centre.

As some of the poor may be delaying or postponing seeking healthcare, a simple logit model might bias the results. Hence, the logit modeling analysis was coupled with a 2-part model: The first part captured the probability of incurring any healthcare expenditure, and the second part estimated the probability of incurring CHE once one seeks healthcare. Table 3 presents the results of the exercise when using 2 thresholds for CHE: 10% and 25%. As with the logit model, here, too, we present the findings for our 2 variables of interest (socioeconomic status and availability of healthcare facilities), while the rest of the findings are presented in Table I in S1 Tables. There are 2 main findings that emerge from the analysis. First, it is evident that those in the lowest consumption quintile are less likely to seek healthcare, but once they do, they are also more likely to experience financial catastrophe. Second, lack of healthcare facilities in the village/ward (or nearby) reduces the probability that people will access healthcare. The results suggest that this is particularly the case with hospitals, whether public or private. Furthermore, additional findings emerge on the other correlates of utilization/CHE: Households that are heavy users of healthcare (e.g., households with children and elderly) are more likely to suffer CHE; by contrast, households with a head who is employed are less likely to incur CHE. Finally, there is some scant evidence linking the educational attainment of the head of household with the probability of using healthcare and, subsequently, incurring CHE.

**Table 3:** Myanmar, determinants of CHE, Sartori model, coefficients, and 95% confidence interval.

CHE threshold: 10%			CHE threshold: 25%		
	Selection	Outcome		Selection	Outcome
**Socioeconomic status** (relative to the highest consumption quintile)			**Socioeconomic status** (relative to the highest consumption quintile)		
	
Quintile 1	−0.395*** [−0.50 to −0.28]	0.08* [−0.005 to 0.18]	Quintile 1	−0.399*** [−0.51 to −0.28]	0.147** [0.02 to 0.27]
Quintile 2	−0.079 [−0.18 to 0.03]	0.017 [−0.06 to 0.10]	Quintile 2	−0.076 [−0.18 to 0.03]	−0.008 [−0.12 to 0.10]
Quintile 3	0.101* [−0.006 to 0.21]	0.04 [−0.03 to 0.12]	Quintile 3	0.10* [−0.004 to 0.21]	0.006 [−0.10 to 0.12]
Quintile 4	0.05 [−0.044 to 0.153]	0.07* [−0.004 to 0.14]	Quintile 4	0.05 [−0.04 to 0.16]	0.035 [−0.06 to 0.14]
**Availability of healthcare facilities**			**Availability of healthcare facilities**		
Public hospital	−0.233* [−0.49 to 0.02]	−0.32** [−0.61 to 0.04]	Public hospital	−0.23* [−0.49 to 0.02]	−0.291 [−0.68 to 0.09]
Public PHC	−0.06 [−0.15 to 0.03]	−0.019 [−0.09 to 0.06]	Public PHC	−0.06 [−0.15 to 0.03]	−0.027 [−0.13 to 0.08]
Private hospital	−0.126** [−0.22 to −0.03]	0.05 [−0.03 to 0.13]	Private hospital	−0.13** [−0.22 to 0.03]	0.093* [−0.016 to 0.20]
Private PHC	−0.078 [−0.17 to 0.02]	−0.07 [−0.15 to 0.01]	Private PHC	−0.07 [−0.17 to 0.03]	−0.019 [−0.13 to 0.009]
Observations	13,730	13,730		13,730	13,730

*** Significant at 1%.

** Significant at 5%.

* Significant at 10%.

The models also control for the following variables: age of the household head, gender of the household head, education attainment of the household head, employment status of the household head, household size, number of household members over the age of 65, number of household members below the age of 5, urbanicity (urban/rural residence) as well as regional dummies for the 15 states/regions in Myanmar. Availability of healthcare facility is derived from the community questionnaire, and it is a binary variable taking a value of 1 if there is no specified healthcare facility in the village/ward or the community does not use such facilities as they are too far. Full set of results is reported in Table I in S1 Tables. Quintile 1 is the lowest, while quintile 4 is the highest proxy for socioeconomic status.

Source: MLCS 2017 and authors’ calculations.

CHE, catastrophic healthcare expenditure; MLCS, Myanmar Living Conditions Survey; PHC, primary healthcare centre.

In order to shed further light on the link between CHE and its main correlates, an additional exercise was conducted involving simple correlation between state/regional level CHE on one hand and demand and supply side variables on the other (the results are reported in Table J in S1 Tables). Overall, the results suggest a slightly higher correlation between CHE and demand/household level variables, in particular, households with higher share of members over the age of 65 tend to be linked with higher incidence of CHE.

## Discussion

This analysis, to the best of our knowledge, is the first of its kind to offer a comprehensive insight into the barriers slowing Myanmar’s progress toward UHC. A significant heterogeneity across states and regions was found, with a higher UHC index found in states with a lower poverty headcount. More broadly, poverty and socioeconomic status were identified as the main driving forces behind unequal access to the interventions that are crucial for achievement of UHC in Myanmar by 2030. In addition, we found that the lack of healthcare infrastructure also contributes to unequal access to the selected healthcare interventions. Finally, our financial risk protection analysis supports the notion that the poor are less likely to use healthcare services and that, once they do, they are at a greater risk of suffering financial catastrophe.

Our findings place Myanmar’s overall UHC index at 65.4, with significant heterogeneity across regions/states. The pattern of correlation between the UHC index and poverty headcount rate follows the pattern previously established in cross-country regression analyses that have demonstrated that regions that are well off also fare much better on the UHC index [4]. In addition, the UHC index is higher in states that, as mentioned in the introduction, have higher density of healthcare facilities (e.g., Yangon or Nay Piy Taw). Notably, the UHC index is higher in states/regions (e.g., Kayah), which could be due to healthcare being delivered through ethnic-based community organizations [28]. Our UHC index is slightly higher than the one derived by Wagstaff and Neelsen as we do not include cancer screening indicators in our analysis, given the unavailability of recent subnational data for these [4].

We also found a pro-rich inequity in access to both prevention and treatment with significant heterogeneity across states/regions. In that respect, Myanmar is comparable to various middle-income countries where there is a consistent pro-rich inequity in access to healthcare, regardless of whether the healthcare is curative or preventative. In the context of the Middle East, for example, a recent UN Children’s Fund report found a pro-rich inequity in most maternal and child health interventions (e.g., 4 antenatal care visits and skilled assistance during delivery) across lower middle-income and upper middle-income countries in the region. The report also found that the severity of the pro-rich inequity is particularly pronounced in the poorest countries in the region [29,30]. Similar findings have been documented previously in some of the larger countries in Latin America, as well as in some lower middle-income countries in sub-Saharan Africa [31,32].

Poverty and socioeconomic status are the main contributors to unequal access to selected healthcare interventions. This finding is consistent with that previously established at a national level, as well as findings by small-scale studies for various states/regions. In particular, it has been documented that poor women in coastal and mountainous areas (e.g., Chin, Rakhine, and Shan) have lower access to healthcare services [33,34]. Consistent with our findings on the contribution of women’s level of education to inequity of access, previous studies have found that a lack of women’s empowerment—the result of a variety of factors, such as poor education, ethnicity and religious diversity, linguistic limitations, and cultural and gender norms—contributes to women’s ability to access maternal healthcare services [35].

Urbanicity (our proxy for availability of healthcare services) also contributes to inequity in access to selected healthcare interventions. The density of healthcare facilities is higher in the urban areas (particularly secondary and tertiary care) with the result that women from rural and remote areas must either forgo care completely or seek care from expensive private healthcare facilities. This has been noted as a problem in Chin state and Ayeyarwaddy [13]. In addition to healthcare facilities, the lack of adequately trained staff has been raised as an issue by ethnic minorities and people in remote areas, such as in Mon and Chin [36]. A previous study suggested that the shortage of health workers, especially in hard-to-reach or remote areas, was strongly linked to slow progress in increasing provision of maternal, neonatal, and child health care in Myanmar [37]. Most recently, inadequate healthcare infrastructure, lack of motivation, geographic, and transport barriers have also been pointed out as a main barrier to achieving UHC from a point of view of healthcare providers [38].

Previous studies in Myanmar found a positive correlation between socioeconomic status and probability to incur CHE [33]. Unlike them, we found that the poor are much more likely to incur CHE, consistent with the emerging international evidence in middle-income countries [39–42]. One of the reasons is that the earlier study relied on a survey conducted by the United Nations Development Programme (UNDP) in 2010, whose sampling frame was based on the 1983 census and omitted some of the border areas (due to security problems) [33,43]. Moreover, our findings suggest that the poor are not only less likely to use healthcare but also that, once they do, they are at a higher risk of experiencing financial catastrophe [24]. Previous narratives from qualitative surveys suggest that a lack of healthcare insurance and a lower ability to pay are the main reasons for this strong nexus between poverty and CHE [44]. Moreover, in the absence of the capacity to pay, households often delay the decision to seek healthcare, up to a point where the treatment becomes so expensive that it pushes households below the poverty line [44].

The results also suggest that there is a strong correlation between state/regional level CHE and a higher share of households with elderly members, echoing findings from a smaller survey conducted in selected townships in 8 out of the 15 states and regions in Myanmar [45]. This result is indicative of 2 things: (i) the elderly are heavier users of healthcare; and (ii) in absence of a comprehensive health insurance, the payments for treatment, often expensive, are made out of pocket. By contrast, the correlation between CHE and the share of households with children below the age of 5 (another category of heavy users) is lower. This, in part, reflects the existence of ongoing schemes (e.g., Maternal and Child Health Voucher Scheme) that offer some financial risk protection to this segment of the population [46]. In addition, we found that both the likelihood of seeking healthcare and the probability of incurring CHE are correlated with the availability of healthcare facilities, although to a lesser extent, compared to some of the household level variables mentioned above [44].

## Limitations

Our study has some limitations. Both surveys in our analysis are cross-sectional in nature; hence, the statistical analysis only establishes an association, rather than a causal link. Second, recall bias has been reported as a significant limitation in similar studies. While the MLCS data contain information on availability of healthcare services, no such information is provided in the DHS. We have experimented with using the state/regional distribution of health workers, but this variable is highly correlated with the variable capturing the 15 states/regions in the country and thus has a bearing on its explanatory power. Finally, MLCS does not contain information on the availability of health insurance, preventing us from further studying the insurance/CHE nexus. However, the results suggest that the values of the UHC index are higher in states/regions where most of the government employees reside (e.g., Yangon, Mandalay, and Nay Pyi Taw).

## Conclusions

We assessed Myanmar’s progress toward achieving UHC on a national and subnational basis. We found that poverty is the main driving force behind the low coverage for selected services (e.g., antenatal care, skilled birth assistance, and full immunization). Not only are the poor less likely to seek healthcare, but also, once they do, they face a much higher likelihood of experiencing a financial catastrophe. Moreover, a lack of healthcare facilities worsens healthcare utilization rates.

These study results suggest that a 2-pronged approach may help Myanmar achieve UHC. First, government and state authorities should reduce the financial burden incurred when seeking healthcare. Some positive steps in this direction have already occurred with the introduction of the Maternal and Child Health Voucher Scheme in 2013 [46]. Extending insurance coverage to vulnerable groups would further improve financial risk protection. Two of Myanmar’s immediate neighbors (Thailand and Vietnam) offer good examples of how the introduction of social health insurance can reduce the probability of incurring CHE among the poor and the vulnerable [47]. Second, the development of comprehensive primary healthcare services must be coupled with significant investment in health infrastructure and the health workforce, particularly in poorer and more remote states. Myanmar currently spends 4.1% of GDP on healthcare, of which, as discussed in the introduction of the paper, around 1% is public [8]. As argued by the International Monetary Fund, Myanmar needs a total spending on healthcare (public and private) of around 9.6% of GDP if it is to realize the SDG health agenda by 2030 [48]. It appears that the NHP (2017 to 2021) aligned with these suggestions. Strategically, the plan envisaged significant investments, particularly in primary healthcare. More importantly, the plan was driven by geographic prioritization, emphasizing availability of healthcare services to townships with greatest need. Finally, it also envisaged provision of a basic package of healthcare services, with plans to expand this over time and increase financial protection [12]. However, the empirical literature suggests that military coups (as the one staged in Myanmar on February 1, 2021) are usually accompanied with significant increases in military expenditure that crowds out social spending [49]. To what extent this has already occurred and will continue to do so is yet to be assessed.

## Supporting information

S1 Checklist The RECORD statement—checklist of items, extended from the STROBE statement, which should be reported in observational studies using routinely collected health data. RECORD, REporting of studies Conducted using Observational Routinely collected health Data; STROBE, STrengthening the Reporting of OBservational studies in Epidemiology.
(DOCX)

S1 Tables Supporting information tables. (DOCX)

S1 Text Supporting information text. (DOCX)

S1 Figures Supporting information figures. (DOCX)

## Abbreviations

ART: antiretroviral therapy
CHE: catastrophic healthcare expenditure
CI: concentration index
DHS: Demographic and Healthcare Survey
EPHS: Essential Package of Health Services
GDP: gross domestic product
GP: general practitioner
MLCS: Myanmar Living Conditions Survey
NHP: National Health Plan
PPP: purchasing power parity
RECORD: REporting of studies Conducted using Observational Routinely collected health Data
SDG: Sustainable Development Goal
UHC: universal health coverage
UNDP: United Nations Development Programme
