# Supplementary material for: Evaluation of progress toward universal health coverage in Myanmar: A national and subnational analysis
Source: PLoS Med. 2021 Oct 15;18(10):e1003811. doi: 10.1371/journal.pmed.1003811 (PMC8519424; doi:10.1371/journal.pmed.1003811)
Supplement: S1 Tables — (DOCX) [file pmed.1003811.s002.docx]

**Supplementary Tables**

Table A. UHC index and coverage of selected interventions (in %), national and subnational analysis

Table B. Myanmar: basic demographic variables by state/region

Table C. Original UHC index our adapted UHC index re-created on the original Wagstaff and Neelsen dataset while excluding cervical and breast cancer screening

Table D. Myanmar - Concentration Index (CI) analysis of selected interventions – subnational analysis

Table E. Myanmar - Concentration Index (CI) analysis of selected interventions – subnational analysis

Table F. Myanmar: Decomposition analysis of the Concentration Index (CI) analysis for selected interventions

Table G. Myanmar: Decomposition analysis of the Concentration Index (CI) analysis for selected interventions, including healthcare worker density

Table H. Myanmar: Determinants of catastrophic healthcare expenditure

Table I. Myanmar: Two-part model on determinants of catastrophic healthcare expenditure

Table J. Region/state level correlation between CHE and both, demand and supply related variables

Table A. UHC index and coverage of selected interventions (in %), national and subnational analysis

| UHC index | Dimension's share in the UHC index | Dimensions | Domain's share in the UHC dimensions | Domain | Indicator's share in the respective domain | Indicator | Indicator definition |
| --- | --- | --- | --- | --- | --- | --- | --- |
|  | 50% | Service coverage | 25% | Prevention | 50% | 4 ante-natal care visits | Percentage of most recent births in last five years with at least 4 antenatal care visits (women age 1549 at the time of the survey) |
|  |  |  |  |  | 50% | Full vaccination | Percentage of children age 15-23 months who received (a) Bacillus Calmette– Guérin (BCG) against TB, (b) 3 doses of diphtheria-pertussis-tetanus (DPT)/Pentavalent, (c) 3 doses of polio (excluding polio given at birth), and (d) Measles/Measles-Mumps Rubella (MMR), either verified by vaccination card or by recall of respondent. |
|  |  |  | 75% | Treatment | 16.6% | SBA - skilled birth assistance | Percentage of most recent births in last 5 years attended by any skilled health personnel (women age 15-49 at the time of the survey). SBA includes doctor, nurse, midwife and auxiliary midwife. |
|  |  |  |  |  | 16.6% | Treatment for Acute Respiratory Infection (ARI) | Percentage of children under 5 with cough and rapid breathing in the two weeks preceding the survey who had a consultation with a formal health care provider. |
|  |  |  |  |  | 16.6% | Treatment for diarrhoea | Percentage of children under 5 with diarrhoea symptoms in the two weeks preceding the survey who had a consultation with a formal health care provider. |
|  |  |  |  |  | 50% | Inpatient care use in last 12 months (% of population age 18 and older) | Percentage of population age 18 and older using inpatient care in the last 12 months. |
|  | 50% | financial protection | 100% | Catastrophic expenses | 100% | Catastrophic expenditures at 10% | Percentage of population with out-of-pocket health expenses exceeding 10% of household consumption or income. |

Table B. Myanmar: basic demographic variables by state/region

|  | Total population (in millions) | % of urban population | % of female population | total population below the age of 5 (in millions) | Total population above the age of 65 (in millions) |
| --- | --- | --- | --- | --- | --- |
|  |  |  |  |  |  |
| Kachin | 1.6 | 35.7 | 50.3 | 0.19 | 0.07 |
| Kayah | 0.3 | 23.3 | 50.3 | 0.04 | 0.01 |
| Kayin | 1.3 | 20.9 | 53.2 | 0.18 | 0.09 |
| Chin | 0.5 | 19.8 | 52.8 | 0.07 | 0.03 |
| Sagaing | 4.9 | 16.4 | 52.6 | 0.42 | 0.38 |
| Taninthary | 1.3 | 24.1 | 50.3 | 0.15 | 0.08 |
| Bago | 4.8 | 21.7 | 52.3 | 0.48 | 0.33 |
| Magway | 3.6 | 12.9 | 55.5 | 0.29 | 0.31 |
| Mandalay | 5.6 | 32.1 | 53.6 | 0.44 | 0.53 |
| Mon | 1.7 | 27.8 | 54.6 | 0.19 | 0.17 |
| Rakhine | 2.7 | 12.7 | 52.9 | 0.27 | 0.20 |
| Yangon | 7.1 | 71.1 | 53.3 | 0.57 | 0.54 |
| Shan | 5.3 | 23.3 | 52.0 | 0.61 | 0.31 |
| Ayeyerwady | 5.8 | 12.7 | 53.1 | 0.53 | 0.38 |
| Nay Pyi Daw | 1.0 | 27.6 | 53.0 | 0.09 | 0.06 |
|  |  |  |  |  |  |

Source: MLCS, 2017 and authors’ calculations

Table C. Original UHC index our adapted UHC index re-created on the original Wagstaff and Neelsen dataset while excluding cervical and breast cancer screening

Table D. Myanmar - Concentration Index (CI) analysis of selected interventions – subnational analysis

*Source:* DHS, 2015; MLCS 2017 and author’s calculations.

Table E. Myanmar - Concentration Index (CI) analysis of selected interventions – subnational analysis

*Source:* DHS, 2015; MLCS 2017 and author’s calculations.

Table F. Myanmar: Decomposition analysis of the Concentration Index (CI) analysis for selected interventions

*Source:* DHS, 2015 and author’s calculations. Note: as DHS only interviews women, the years of schooling variable pertains to the years of schooling of the interviewed woman.

Table G. Myanmar: Decomposition analysis of the Concentration Index (CI) analysis for selected interventions, including healthcare worker density

*Source:* DHS, 2015 and author’s calculations. Note: as DHS only interviews women, the years of schooling variable pertains to the years of schooling of the interviewed woman.

Table H. Myanmar: Determinants of catastrophic healthcare expenditure

*Source:* MLCS, 2017 and author’s calculations.

Table I. Myanmar: Two-part model on determinants of catastrophic healthcare expenditure

*Source:* MLCS, 2017 and author’s calculations.

Table J. Region/state level correlation between CHE and both, demand and supply related variables

| Variables | Correlation with CHE at 10% | Correlation with CHE at 25% |
| --- | --- | --- |
| Demand side variables |  |  |
| Poverty headcount | -0.05 | 0.22 |
| Per capita equivalised household consumption | -0.08 | 0.10 |
| Share of households with hh members below the age of 5 | -0.15 | 0.23 |
| Share of households with hh members above the age of 65 | 0.55 | 0.46 |
| Supply side variables |  |  |
| % of communities without public hospital and going to the nearest is deemed ‘too far’ | -0.13 | 0.18 |
| % of communities without public PHC and going to the nearest is deemed ‘too far’ | -0.14 | -0.14 |
| % of communities without private hospital and going to the nearest is deemed ‘too far’ | -0.38 | -0.11 |
| % of communities without private PHC and going to the nearest is deemed ‘too far’ | -0.28 | -0.10 |
| % of communities without a pharmacy and going to the nearest is deemed ‘too far’ | 0.068 | 0.13 |

*Source:* MLCS, 2017 and author’s calculations Note: Table A3 above captures the main demographic variables on a region/state level.
