# Supplementary material for: Evaluation of progress toward universal health coverage in Myanmar: A national and subnational analysis
Source: PLoS Med. 2021 Oct 15;18(10):e1003811. doi: 10.1371/journal.pmed.1003811 (PMC8519424; doi:10.1371/journal.pmed.1003811)
Supplement: S1 Text — (DOCX) [file pmed.1003811.s003.docx]

*UCH index – further details*

As per Wagstaff et al, our UHC index is a geometric mean of the two set of indicators: service coverage (SC) and financial protection (FP) (which as discussed in the main part of the paper is equal to 100-CATA):

𝑈𝐻𝐶 ≡ 𝑆𝐶 ^0.5^𝐹𝑃 ^0.5^ = 𝑆𝐶 ^0.5^ (100 − 𝐶𝐴𝑇𝐴) ^0.5^.

By following this approach, the creation of an index capturing both UHC dimensions allows progress on one UHC dimension to be traded off against progress on the other, as policy makers often are willing to accept worse performance on one dimension (e.g., financial protection) in exchange for better performance on the other (e.g., service coverage) (Wagstaff et al, 2020).

The crucial step in the index creation is the process of selection of the elements of the index. In doing so we were guided by previous studies by Wagstaff et al (2020) as well as Hogan et al (2018) as well as by six broad principles: (i) indicators should be delivered by health providers and should not capture downstream indicators or upstream indicators; (ii) they should be as comprehensive as possible; (iii) they should reflect national priorities as much as they reflect international ones based on the SDG Agenda 2030; (iv) indicators should be based on nationally (and in our case sub-nationally) representative surveys; (v) each indicator should be able to be transformed into a coverage indicator; and (iv) the indicators should be based on available data (Wagstaff et al, 2020).

References:

1. Wagstaff, Adam and Sven Neelsen (2020) “A comprehensive assessment of universal health coverage in 111 countries: a retrospective observational study”, Lancet Global Health, Volume 8, ISSUE 1, e39-e49, January 01, 2020.
2. Hogan DR, Stevens GA, Hosseinpoor AR, Boerma T. Monitoring universal health coverage within the Sustainable Development Goals: development and baseline data for an index of essential health services. The Lancet Global health 2018; 6(2): e152-e68.

*Concentration index for inequality of utilization*

In order to assess the equity of utilization we used the standard concentration index (CI) and decomposition of CI in order to quantify the degree of equality in the utilization of health services and the extent to which our main variables of interest contributed to the inequality of utilization (O’Donnell et al, 2007).

CI is defined with reference to the concentration curve. It is twice the area between the concentration curve and the line of equality (the 45-degree line). Concentration curves plot the specific health variable in the y–axis against the percentage distribution based on a wealth measure in the x–axis. Therefore, CI takes a value ranging from (-1, 1) where negative values express pro-poor concentration and positive values express pro-rich concentration. Equation B presents the general model for CI:

$C=\frac{2}{\mu} {cov}_{w}(y_{i}, r_{i})$ (B)

Where *C* is the CI, *y*_i_ is the measure of utilization of healthcare services, µ is its mean, and *r_i_* is the rank distribution of an individual *i* according to his wealth index.

The decomposition of the CI shows the contribution of the independent variables in the logit model to the distribution (inequality) of health services based on the wealth rank of the population. It provides more detailed information and raises potential areas for policy intervention. We relied on methodology for the decomposition analysis that used a probit model and its ‘partial effects’ (i.e. the effects of an individual independent variable, ceteris paribus) as equation C depicts:

$E\left( y_{i} | x_{i} \right)=G \left( \sum_{k} \beta_{k}x_{k}^{i} \right)$ (C)

where G represents the functional form for a nonlinear model. As proposed by van Doorslaer et al (2004), we have restored the mechanics of the decomposition framework by replacing the βk parameters in the equation with the βmk parameters, where the βmk represent the partial effects of the x (the determinants of y) in the linear approximation of the non-linear model expressed by Equation (D):

$y_{i}= \sum_{k} \beta_{k}^{m}x_{k}^{i}+ \mu_{i}$ (D)

Accordingly, we conducted a decomposition of the socio-economic related inequality affecting healthcare utilization of our main variables of interest. For the purpose of this paper, we conducted the decomposition analysis for the set of indicators exhibiting pro-rich inequity. The choice for the set of independent variables used in the decomposition analysis was based on the availability of data in the DHS. As such, the battery of independent variables is able to capture enabling factors (e.g. educational attainment, wealth index and age) as well as community level factors (urbanicity and region of residence).

The education attainment is captured by a continuous variable reporting the number of years of schooling. The wealth index is the standard categorical variable with 5 categories included in the DHS (poorest, poorer, average, richer, richest). The respondent is a continuous variable capturing the age (in years) of the respondent. Finally, there are two additional variables used as proxy for community level variables: urbanicity (a dummy variable capturing if the respondent lives in an urban or rural setting) and region (a categorical variable capturing the 15 different states/regions in Myanmar).

References:

1. O’Donnell O, Van Doorslaer E, Wagstaff A, Lindelow M. Analyzing health equity using household survey data: a guide to techniques and their implementation. The World Bank; 2007.
2. van Doorslaer E, Koolman X, Jones AM. Explaining income-related inequalities in doctor utilization in Europe. Health Econ. 2004;13:629–47.

*Catastrophic healthcare expenditure, determinants of catastrophic healthcare expenditure and Sartori model on determinants of CHE*

***Catastrophic healthcare expenditure***

In order to derive the CHE (catastrophic healthcare expenditure) we rely on the standard approach, i.e. we estimate the share of households experiencing catastrophic healthcare expenditure. Let $N$ the number of households, and $E$ a binary indicator that takes the value of 1 if ${oop}_{i}/{exp}_{i}$ is greater than the respective threshold $z$, and 0 otherwise. The headcount can be thus expressed as follows:

$$Headcount=\frac{1}{N}\sum_{i=1}^{N} E_{i}$$

Where oop is the out of pocket healthcare expenditure and exp is the total household expenditure. In creating the total oop we rely on both, the individual and the household modules of the MLCS survey. Data from the individual module include the following oop categories: expenditure on outpatient care, expenditure on inpatient care, expenditure on transport when seeking healthcare and expenditure on overnight stays when seeking healthcare. From the household module of the survey we take the data on expenditures on pharmaceuticals. The final consumption expenditure has been previously derived and the results of the process are reported elsewhere (CSO, Poverty report, 2019).

Finally in calculating the CHE we use four different thresholds: 10%, 15%, 20% and 25% as a share of total household consumption. There exist other metrics that capture CHE (e.g. capacity to pay approach), which calculate the catastrophic payments as a share of total expenditure minus spending on food (see Wagstaff (2019)). However, as these measures have received some criticism recently, we do not include them in this analysis.

***Determinants of CHE***

As per the existing literature, we commence our analysis using the standard logit modelling. In doing so we use the thresholds above to create four different CHE binary variables which are used as dependent variables in our modelling efforts. Against this background, if we assume a linear model, the probability of incurring CHE would be analyzed by regressing the CHE variable (y_i_) on a vector of variables capturing household (x), community (u) and regional (z) characteristics.

The equation would be as follows:

$y_{i}^{*}=\alpha+\sum_{k} \beta_{k}x_{k,i}+\sum_{q} \delta_{q}u_{q,i}+ \sum_{p} \gamma_{p,i}z +\varepsilon_{i}$ , with i = 1,…N *(A)*

Where α, β, γ_,_ δ= parameters and ε_i_ = error term.

Assuming that y_i_^*^ in equation (A) is a latent variable, the logit model is written as:

$$\left\{ \begin{aligned} 1 if y_{i}^{*}>0 \\ 0, otherwise \end{aligned} \right.$$

We use the following set of characteristics in our modelling efforts.

Characteristic of the household. We use a few variables to capture the characteristics of the household: (i) a dummy variable capturing if the household is headed by a male or a female; (ii) a set of dummy variables for the age of the household head: less than 30, 30 to 39, 40 to 49, 50 to 59, 60 to 69, 70 to 79 and over 80; (iii) employment status of the household head captured by a dummy variable if the household head is employed or unemployed; (iv) marital status of the household head; (v) disability status of the household head; (vi) medical need variables captured by the number of household members less than 5 years or age as well as number of household members over the age of 65; (vii) residence of the household captured by a urban/rural dummy variable.

Characteristics of the community. As evidenced by the previous research (Xu et al, 2003), in addition to the capacity to pay, the availability of healthcare services also contributes to the probability of incurring catastrophic healthcare expenditures. Hence and in order to capture the availability of healthcare services, we rely on the community module of the MLCS survey. The module, inter alia, includes questions on availability of various services in village/ward level (e.g. schools, healthcare facilities, banks etc). Based on these questions we have constructed four dummy variables that capture the availability of healthcare services (both primary and secondary, public and private) at a community level: (i) distance to public hospital (there is no public hospital in the village/ward and the community doesn’t use it because it’s far); (ii) distance to public primary healthcare centre (there is no public primary healthcare centre in the village/ward and people do not use this type of healthcare facilities because they are too far. The following were considered as primary healthcare facilities: government health centre (RHC), government health post, government stand-alone VCT centre, government family planning clinic); (iii) distance to a private hospital (there is no private hospital in the village/ward and people don’t use it because it’s too far); (iv) distance to private primary healthcare centre (there is no primary private healthcare centre in the village/ward and people do not use it because it’s too far. The following were considered as primary private healthcare facilities: private single doctor’s office/clinic, private stand-alone VCT clinic).

Characteristics of the regions. Finally we include a set of dummies capturing the regional characteristics of each of the 15 states/regions in Myanmar.

***Sartori selection model: technical details***

In our analysis, we address potential selection issues, arising from the fact that some households may postpone health payments and may not use health care due to financial restrictions and barriers to access (Brown et al, 2014; Kawabata et al, 2002). In doing so, we employ a maximum likelihood estimator proposed by Sartori (2003), which relies on a two-step approach that distinguishes those who seek medical care (Sartori, 2003). Generally speaking, the selection equation models the health care use, while the outcome equation focuses on the probability of facing CHE adjusted for the selection issues (Brown et al, 2014). This approach can be employed in cases of binary outcomes of interest, does not require a different set of regressors for the two equations, and relies on different assumption about the error terms in the two equations. In particular, the selection and outcome equations are given by the following expressions:

Selection equation: $U_{1i}=X_{i}\gamma+\varepsilon_{1i}$

Outcome equation: $U_{2i}=X_{i}\delta+\varepsilon_{2i}$

where $U$ corresponds to the unobserved continuous dependent variables, and $\gamma$ and $\delta$ are the vectors of the regression coefficients for the selection and the outcome equation respectively. Last, $\varepsilon_{1i}$ and $\varepsilon_{2i}$ are normally distributed residual terms.

Given that $U$ is unobserved, the observed outcomes are the following:

$$Z_{1i}=\left\{ \begin{aligned} 0 if U_{1i}<0 \\ 1 if U_{1i}\geq0 \end{aligned} \right.$$

$$Z_{2i}=\left\{ \begin{aligned} 0 if U_{2i}<0 \\ 1 if U_{2i}\geq0 \end{aligned} \right.$$

where $Z_{1i}$ equals 1 if the household $i$ uses health care, and $Z_{2i}$ indicates whether the household $i$ incurred CHE (i.e. the observed outcome of the equation of interest).

Following the approach proposed by Sartori (2003), we further define the following random variables $Y_{ij}$:

$$Y_{i0}=\left\{ \begin{aligned} 1 if Z_{1i}=0 \\ 0 otherwise \end{aligned} \right.$$

$$Y_{i1}=\left\{ \begin{aligned} 1 if Z_{1i}=1 and Z_{2i}=0 \\ 0 otherwise \end{aligned} \right.$$

$$Y_{i2}=\left\{ \begin{aligned} 1 if Z_{1i}=1 and Z_{2i}=1 \\ 0 otherwise \end{aligned} \right.$$

In particular, $Y_{i0}$ is unity if the household does not seek health care (i.e. not selected). Additionally, $Y_{i1}$ equals 1 if the household seeks medical care and the main dependent variable is 0 (i.e. the households does not face CHE), whereas $Y_{i2}$ equals 1 if the household uses health care and the outcome variable equals 1 (i.e. the household incurs CHE). Following the definitions of the random variables $Y_{ij}$, the probability that $Y_{ij}$ takes the value of 1, $\Pr\left( Y_{ij}=1 \right),$ is defined as follows:

$$\Pr\left( Y_{i0}=1 \right)=\Phi\left( -X_{i}\gamma\right)$$

$$\Pr\left( Y_{i1}=1 \right)=\left\{ \begin{aligned} \Phi\left( -X_{i}\delta\right)-\Phi\left( -X_{i}\gamma\right) if \left( \gamma-\delta\right)X_{i}>0 \\ 0 if \left( \gamma-\delta\right)X_{i}\leq0 \end{aligned} \right.$$

$$\Pr\left( Y_{i2}=1 \right)=\left\{ \begin{aligned} \Phi\left( X_{i}\delta\right) if \left( \gamma-\delta\right)X_{i}>0 \\ \Phi\left( X_{i}\gamma\right) if \left( \gamma-\delta\right)X_{i}\leq0 \end{aligned} \right.$$

where $\Phi$ is the cumulative standard normal distribution function.

References:

1. Brown, S., Hole, A.R., Kilic, D., 2014. Out-of-pocket health care expenditure in Turkey: Analysis of the 2003–2008 Household Budget Surveys. Econ. Model. 41, 211–218.
2. Myanmar Living Condition Survey 2017 Report 3 - Poverty Report (English). Washington, D.C.: World Bank Group. <http://documents.worldbank.org/curated/en/921021561058201854/Myanmar-Living-Condition-Survey-2017-Report-3-Poverty-Report>
3. Sartori, A.E., 2003. An Estimator for Some Binary-Outcome Selection Models Without Exclusion.
4. Kawabata K, Xu K, Carrin G. Preventing impoverishment through protection against catastrophic health expenditure. Bull World Health Organ 2002; 80: 612.

Wagstaff Adam (2019). Measuring catastrophic medical expenditures: Reflections on three issues. Health Economics. 2019;28(6):765–781.

1. Wagstaff A. Measuring catastrophic medical expenditures: Reflections on three issues. Health Economics. 2019;28(6):765–781
