# Supplementary material for: Evaluation of progress toward universal health coverage in Myanmar: A national and subnational analysis
Source: PLoS Med. 2021 Oct 15;18(10):e1003811. doi: 10.1371/journal.pmed.1003811 (PMC8519424; doi:10.1371/journal.pmed.1003811)
Supplement: S1 Figures — (DOCX) [file pmed.1003811.s004.docx]

Figure A. Correlation between UHC index and poverty rate in Myanmar, using CHE threshold of 25%


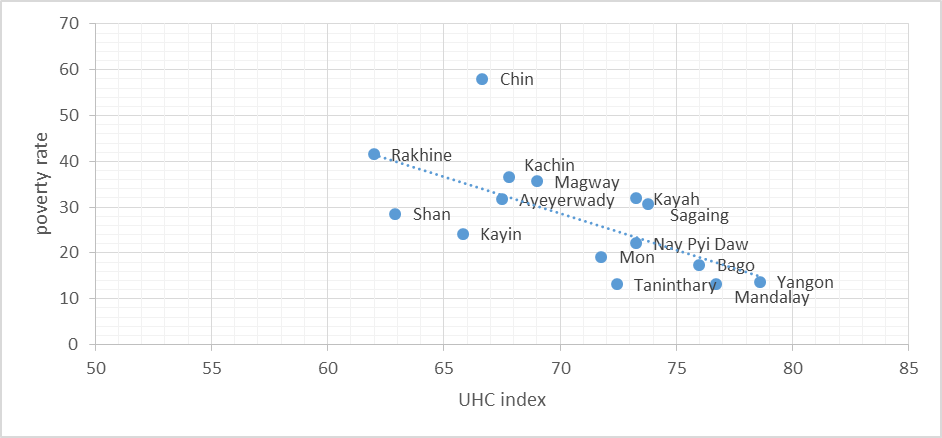


*Source:* DHS, 2015 and MLCS, 2017 and author’s calculations. Correlation coefficient between UHC index and poverty rate is -0.
